# Annual Prevalence, Health Expenditures, and Co-Morbidities Trend of Iron Deficiency Anemia in Korea: National Health Insurance Service Data from 2002 to 2013

**DOI:** 10.3390/ijerph17124433

**Published:** 2020-06-20

**Authors:** Yoo-Jin Park, Hee-Sook Lim, Tae-Hee Kim

**Affiliations:** 1 Department of Interdisciplinary Program in Biomedical Science, Soonchunhyang University Graduate School, Asan 31538, Korea; qkrdbwls0932@naver.com; 2 Department of Food Sciences and Nutrition, Yeonsung University, Anyang 14011, Korea; limhs@yeonsung.ac.kr; 3 Department of Obstetrics and Gynecology, Soonchunhyang University Bucheon Hospital, Soonchunhyang University College of Medicine, Bucheon 14584, Korea

**Keywords:** anemia, iron-deficiency, comorbidity, epidemiology, health expenditures, malnutrition

## Abstract

Despite improvements in nutritional status, iron deficiency anemia (IDA) remains a debilitating nutritional problem worldwide. We estimate annual IDA prevalence rates by sex and age and the trends therein in Korea. We also calculate the health expenditures of IDA and its co-morbidities by analyzing claims data in the National Health Information Database from 2002 to 2013. All analyses were performed based on diagnosis codes of IDA (D50, D50.0, D50.8, and D50.9) regardless of whether IDA was the principal or a coexisting disease. Trends in IDA prevalence rates were evaluated by calculating annual percent changes (APCs) in prevalence. The health expenditures of IDA were calculated based on the direct medical costs (outpatient and hospitalization costs, pharmaceutical costs) and direct non-medical costs (travel costs). The overall IDA prevalence in both sexes increased approximately 2.3-fold from 2002 to 2013; the APC was +7.6%. In females, the prevalence of IDA was highest in aged 30–39 and 40–49 years. The APC was highest in those aged <10 years (+18.2%), followed by those aged ≥80 (+14.7%) and 70–79 (+9.8%) years. In males, the prevalence rates were highest in aged <10 years, followed by those aged ≥60 years. The APC was highest in those aged <10 years (+19.1%), followed by those aged ≥80 years (+10.5%). The total health expenditures increased 2.8-fold during 12 years. Diseases of the respiratory or gastrointestinal tract were the most prevalent co-morbidities in both males and females. The annual prevalence of IDA continues to rise in association with adverse health expenditures and co-morbidities in spite of improvements in nutritional status. Most importantly, infants and young children, the elderly, and females aged 30–49 years are at highest risk of IDA. A national, prospective, and well-organized effort to improve iron status and to manage IDA is required.

## 1. Introduction

Regarding public health, anemia or iron deficiency anemia (IDA) is one of the indicators of nutritional deficiency status. Anemia is defined as a decrease in red blood cell numbers or the hemoglobin level, which in turn reduces the oxygen-carrying capacity of blood [1]. The worldwide prevalence of anemia has been estimated at 48% in preschool-aged children, 25% in school-aged children, 13% in males aged 15–59, 42% in pregnant women, 30% in reproductive aged women, and 24% in the elderly [2]. The causes of anemia include infections (e.g., tuberculosis, malaria, HIV), micronutrient deficiencies (e.g., of iron, folate, and vitamins A/B12/C), and genetic conditions (e.g., sickle-cell disease and thalassemia).

Almost 50% of all cases of anemia are attributable to iron deficiency, which is the most prevalent nutritional deficiency, affecting an estimated 2 billion people worldwide [3]. In South Korea, the prevalence of iron deficiency in females is 17.2%, 24.1%, 33.0%, and 5.7% in ages 10–14, 15–17, 18–49, and ≥70 years, respectively. In males, the prevalence is 8.6%, 3.9%, and 2.6% in ages 10–14, 15–17, and ≥70 years, respectively [4]. Iron deficiency anemia (IDA) not only causes adverse health outcomes, such as weakness (impairments in innate immunity/cell-mediated immunity) [5], poor physical growth, and increased risks during pregnancy (preterm delivery and inferior neonatal health) [6], but also reductions in the work capacity and productivity of both adolescents and adults [7].

In 2010, the overall IDA prevalence was 4.3% in Korea (0.7 and 8.0% in males and females, respectively) [8]. A total of 31.4% of the population with iron deficiency was found to be pregnant women, and the prevalence in elderly women was higher than that of men [9]. In particular, it was observed that women in childbearing age were underweight and were often accompanied by dietary iron deficiency [10]. However, the IDA prevalence varies by sex and age, being affected by various factors. No large-scale nationwide study has yet been performed in Korea, and we analyzed long-term trends in IDA using national claims data. The present study is a population-based nationwide study of the epidemiological burdens imposed by IDA. We estimate annual IDA prevalence rates by sex and age and the trends therein (annual percentage changes, APCs). We also calculate the health expenditures of IDA and its co-morbidities by analyzing claims data in the National Health Information Database (NHID).

## 2. Materials and Methods

### 2.1. Study Subjects

Our institutional review board approved our data collection and analysis (approval no. SCHBC 2015-09-001-004). The annual prevalence, health expenditures, and co-morbidities associated with IDA were investigated using nationwide NHID data from 2002 to 2013. IDA was diagnosed based on the International Classification of Disease 10th Revision; we included all relevant codes (D50 Iron deficiency anemia, D50.0: Iron deficiency anemia secondary to blood loss (chronic), posthemorrhagic anemia (chronic), D50.8: Iron deficiency anemia, and D50.9: Iron deficiency anemia, unspecified) regardless of whether IDA was the principal, or a coexisting, disease. The numbers of the sample cohort population were as follows (Table 1).

### 2.2. Data Resources

#### 2.2.1. The National Health Information Database

We used data from the NHID of the National Health Insurance Service (NHIS), which covers all Korean citizens under two major programs of NHI and Medical Aid (MA). Almost all individuals pay a proportion of their healthcare costs; the proportion varies by institution and type of care. Under MA, the government pays all medical expenses of low-income households who cannot pay for healthcare. Commencing in 2004, MA was expanded to include patients with rare, inveterate, and chronic diseases, and all children aged <18 years [11]. In total, 97% of Koreans are covered by the NHIS and 3% by MA [12]. The NHID thus contains data on the entire Korean population, facilitating population-based nationwide studies that can precisely determine disease patterns. The NHID contains data on the claims of medical providers, including diagnoses, treatments, and services rendered, as well as on the inpatient and outpatient drugs prescribed [13].

#### 2.2.2. The Korean National Health and Nutritional Examination Survey (KNHANES)

The KNHANES is a national surveillance system assessing the health and nutrition of Koreans. The surveys are implemented by the Korea Centers for Disease Control and Prevention, as mandated in the National Health Promotion Act. For the KNHANES, nationwide cross-sectional studies have been performed annually since 1998. The KNHANES yields objective, standardized data on disease epidemiology, socioeconomic status, health behaviors, and healthcare service utilization; these basic data inform national healthcare policies [14]. The two-way travel costs of outpatients and inpatients are also captured. Here, we use the daily travel costs when visiting clinics as reported in KNHANES IV (2009), V (2010–2012), and VI (2013).

#### 2.2.3. Korean Statistical Information Service (KOSIS)

Statistics Korea (KOSTAT) makes various statistical data available through KOSIS, which published 86,221 statistical tables covering 756 topics in 2015. The population data (including those by sex and age) were applied herein to calculate IDA prevalence.

#### 2.2.4. Prevalence of IDA

The annual prevalence of IDA was calculated by dividing the number of cases by the annual, residence-registered mid-year population, as recorded by KOSIS [15]. Cases of recurrence associated with retreatment during the survey year were regarded as new cases; if any such case continued into the following year, it was excluded from that later year. Prevalence rates by sex and age were derived by calculating age-standardized rates (ASRs) from 2002 to 2013. Furthermore, trends in IDA prevalence rates were evaluated by calculating APCs in prevalence [16]:

(1) $$ASRs=\frac{\sum a_{i}w_{i}}{\sum w_{i}}$$

where *a_i_* is the age-specific rate of the *i*-th age group and *W_i_* is the size of the *i*-th age group in the standard population.

(2) $$APC=(e^{b}-1)\times 100$$

where *b* is the slope of the regression of the natural logarithm of the ASR in that year.

#### 2.2.5. Health Expenditures of IDA

The health expenditures of IDA (direct medical and non-medical costs) were evaluated using the NHID, KNHANES, and KOSIS databases. We calculated outpatient and hospital costs (paid by both the insurer and patients), the costs of pharmaceuticals obtained in hospitals/pharmacies, and travel costs, using formulae reported previously [17,18].

#### 2.2.6. Direct Medical Cost

Direct medical costs were calculated with NHID data paid by the insurer or patient. Direct medical costs include outpatient and hospitalization costs and pharmaceutical costs. Patients paid 20.5% of their clinic/hospital costs and 2.9% of their pharmaceutical costs [16,19].

(3) $$\text{Direct medicial cost}=\sum_{i}\sum_{j}\left(\frac{OE_{ij}}{1-\alpha }\right)+\frac{E_{ij}}{1-\beta }+\frac{ED_{ij}}{1-\gamma }$$

(4) $$\mathrm{where}\left\{ \begin{array}{l} i=0,1,\ldots ,\mathrm{age}\\ j=1,2,\mathrm{sex}\\ \alpha =\text{proportion of non-insured costs paid by patients for visiting clinics}\\ \beta =\text{proportion of non-insured costs paid by patients for hospitalization}\\ \gamma =\text{proportion of non-insured costs paid by patients for buying medicines}\\ {OE}_{ij}=\text{amonut claimed for visting clinics}\\ E_{ij}=\text{amount claimed for hospitalization}\\ {ED}_{ij}=\text{amount claimed for buying medicines} \end{array}\right.$$

#### 2.2.7. Direct Non-Medical Cost

Direct non-medical costs included travel expenses to and from the outpatient clinic or hospital. The average round-trip expenses were 1.25 United States Dollars (USD) and 5.64 United States Dollars, respectively [20]. These costs were multiplied by the annual inflation rate recorded by KOSIS. Those aged <20 or >64 years were assumed to visit with a guardian, and their travel costs were thus doubled [16].

#### 2.2.8. Co-Morbidities of IDA

The 10 most frequent IDA co-morbidities were ranked in both males and females.

### 2.3. Statistical Analyses

The APCs of IDA were calculated using linear regression to allow evaluation of trends. The prevalence rates of co-morbidities were estimated via linear-by-linear association. All statistical analyses were performed using SPSS (ver. 24.0; IBM Corp., Armonk, NY, USA) and R (ver. 3.1.3; R Foundation for Statistical Computing, Vienna, Austria) software, and a two-tailed *p*-value < 0.05 was considered significant.

## 3. Results

### 3.1. Prevalence of IDA

**Figure 1:**
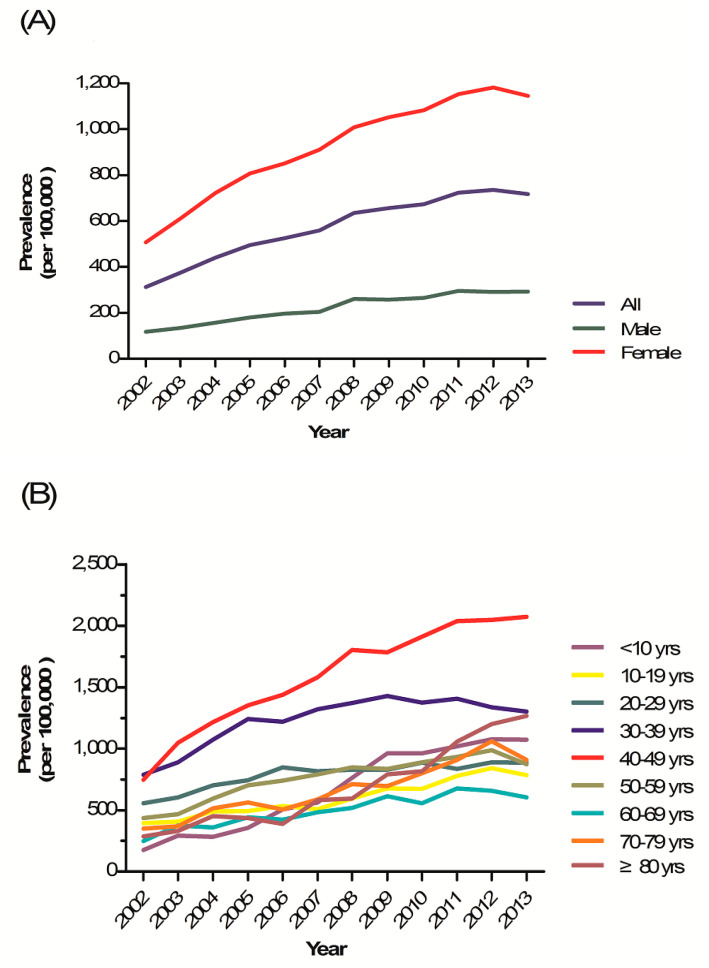

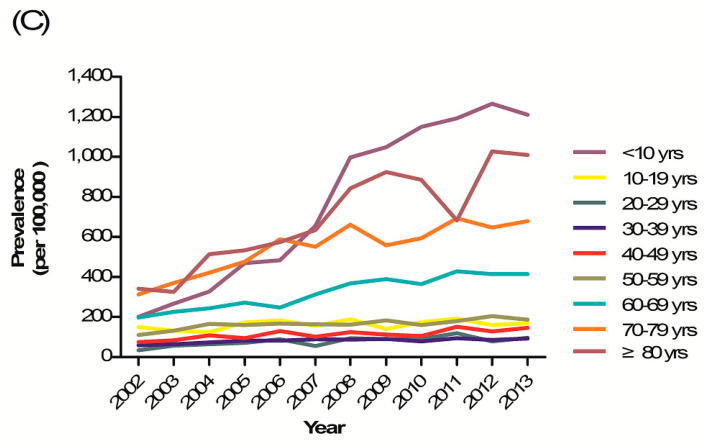
Tendency of iron deficiency anemia prevalence in Korea, 2002–2013. (**A**) Prevalence of iron deficiency anemia in the whole population. (**B**) Prevalence of iron deficiency anemia in females. (**C**) Prevalence of iron deficiency anemia in males.

Growing children and pregnant and lactating women have the highest demand of Fe and bioavailability is severely compromised in old age. The prevalence rate by sex and age were as follows (Figure 1, Table 2, Table 3).

#### 3.1.1. Prevalence by Sex

The overall IDA prevalence in both sexes increased approximately 2.3-fold from 2002 to 2013; the average APC was +7.6%. The prevalence in females increased 2.3-fold over the 12-year period and was always higher than in males. The IDA prevalence in males increased 2.5-fold during the same period. The APC of males (+8.9%) was higher than that of females (+7.4%) (Figure 1, Table 2).

The female-to-male prevalence ratios (PRRs) from 2002 to 2013 were ≥1 in subjects in the second-to-seventh decades of life. On the contrary, the PRRs in children aged <10 years were <1 in all years, and IDA was more prevalent in males than females. The PRRs in those aged ≥80 years were <1 until 2010, but reversed in 2011, becoming more prevalent in females thereafter (Table 3).

#### 3.1.2. Prevalence by Age

The prevalence of IDA was highest in females aged 30–39 and 40–49 years. The APC was highest in those aged <10 years (+18.2%), followed by those aged ≥80 (+14.7%) and 70–79 (+9.8%) years. (Figure 1B, Table 2)

The prevalence of IDA in males increased over the period 2002–2013 in all age groups. The prevalence rates were highest in infants, toddlers, preschool children aged <10 years, and those aged ≥80 years. The APC was highest in those aged <10 years (+19.1%), followed by those aged ≥80 years (+10.5%) (Figure 1C, Table 2).

### 3.2. Health Expenditures of IDA

#### 3.2.1. Total Treatment Costs

**Figure 2:**
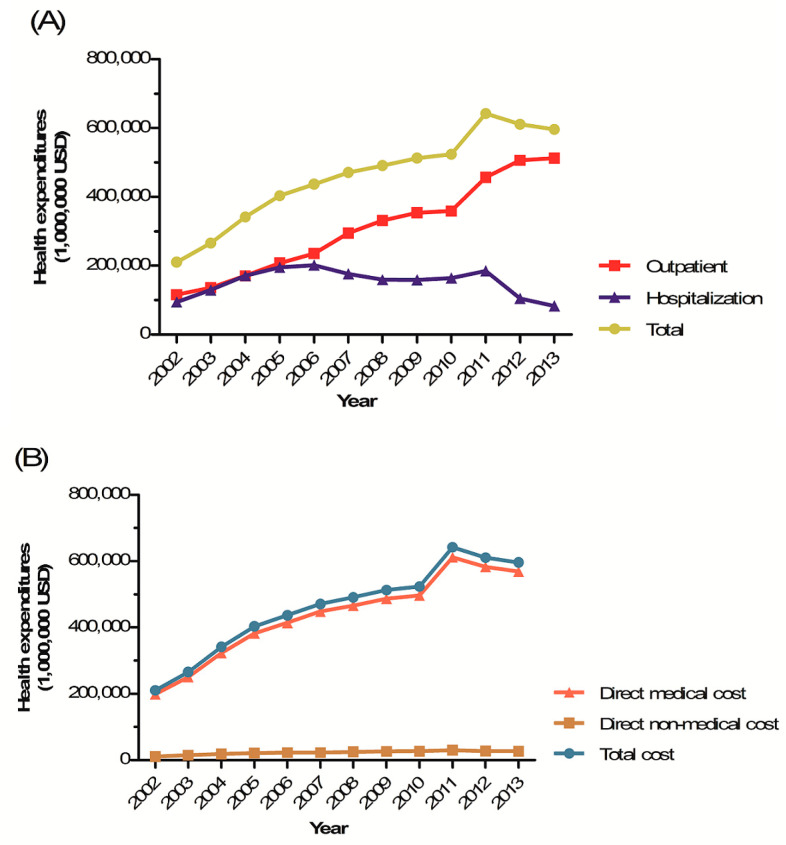
Health expenditures of iron deficiency anemia in Korea, 2002–2013. (**A**) Health expenditures of iron deficiency anemia in accordance with service types. (**B**) Health expenditures of iron deficiency anemia in accordance with cost types.

The total health expenditure increased 2.8-fold from $210,203 in 2002 to $596,256 in 2013. Outpatient treatment became more common over time, and hospitalization less so. The gap between outpatient and hospitalization costs widened after 2005; the total outpatient cost in 2013 was 6.2-fold that of hospitalization. The hospitalization costs per capita were higher than the outpatient costs in all years. The per-capita cost of hospitalization increased notably from 2012 (Figure 2A, Table 4).

#### 3.2.2. Direct Medical Costs

The total direct medical cost increased approximately 2.9-fold over the 12-year study period. The costs incurred by both insurers and patients were greater than those of pharmaceuticals prescribed in either hospitals or pharmacies, and this gap was wider in 2013 than 2002 (Figure 2B, Table 4).

#### 3.2.3. Direct Non-Medical Costs

Total, direct non-medical costs increased 2.4-fold from $11,221 in 2002 to $27,099 in 2013. The direct non-medical costs for outpatients increased 5.5-fold from $4760 in 2002 to $26,093 in 2013, but hospitalization costs decreased 6.4-fold from $6461 in 2002 to $1005 in 2013 (Figure 2B, Table 4).

### 3.3. Co-Morbidities

**Figure 3:**
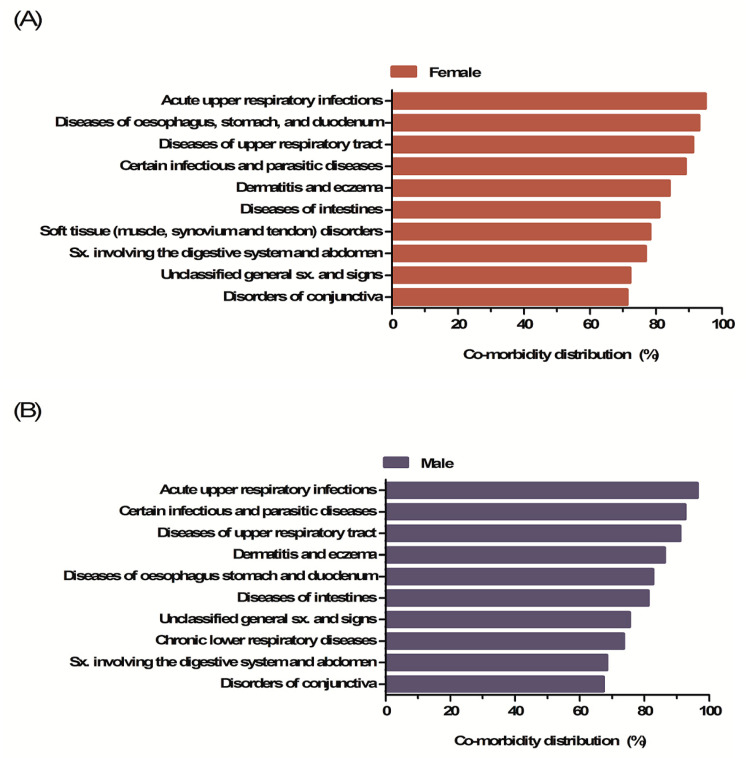
Co-morbidities of iron deficiency anemia. (**A**) Co-morbidities of iron deficiency anemia in females. (**B**) Co-morbidities of iron deficiency anemia in males.

Acute upper respiratory tract infections (URIs) were the most prevalent comorbidity in both females (95%) and males (96.4%). For male, gastrointestinal diseases (94.2%) were ranked second and other upper respiratory problems (93.0%) were ranked third. In the case of females, infectious diseases (92.5%) were ranked second, and other upper respiratory diseases (91.4%) were ranked third. Dermatitis and eczema (84% and 86.2%), and disorders of the conjunctiva (71.3% and 67.3%), were the next most common comorbidities in females and males, respectively (Figure 3).

## 4. Discussion

We present interesting insights into annual changes in IDA prevalence from 2002 to 2013 by sex and age, and the associated health expenditures and co-morbidities. Despite improvements in public health facilities and the development of iron-fortified foods, the prevalence of IDA is still at a level of public health concern in all sexes and ages. Notably, annual IDA prevalence has increased most rapidly over the last 12 years in those aged <10 years, followed by those aged ≥80 and 70–79 years, of both sexes. Females aged 30–49 years are also at highest risk of IDA. The health expenditures of IDA have also increased; IDA was commonly accompanied by diseases of the respiratory or gastrointestinal tract.

The overall IDA prevalence increased in both males and females from 2002 to 2013. The greatest increase was evident in those aged <10 years, probably caused by inappropriate feeding and growth spurts in infants and children, and/or the mother’s low iron status during pregnancy. Infants aged ≥6 months cannot obtain sufficient iron via breastfeeding alone; they lack iron stores adequate for growth spurts [21]. Generally, breastfed infants require iron-fortified weaning when aged ≥ 2 months. However, many homemade weaning formulae have low iron contents [22]. Furthermore, the early use of cow’s milk may explain the rapid increase in IDA prevalence. Such milk is recommended for those aged >14 months; however, 6.6% of Korean infants are fed cow’s milk when aged <12 months [23,24]. IDA can also be prevalent in children. The KNHANES found that the iron intake of preschool children was 7.0 mg/day, 79.8% below the recommended level, explaining the high risks of IDA in infants and children in Korea [25]. Furthermore, the prevalence of anemia ranges from around 20–30% in pregnant women in Korea [26,27]. Maternal iron deficiency may contribute to low iron stores during infancy, causing an increase in IDA in infants and toddlers if not treated properly.

The IDA prevalence also increased notably in the elderly. The KNHANES III (1988–1994) surveyed >5000 community-dwelling elderly subjects. In the USA, 11.0% of males and 10.2% of females aged ≥65 years were anemic [28]. Of the various anemias, IDA was the most common (25.4%) [29]. The marked increase in IDA prevalence in those aged ≥ 80 years may be associated with chronic diseases and/or poor dietary habits. The prevalence of chronic diseases that are attributable to IDA in the elderly is increasing in Korea. IDA is often accompanied by chronic inflammatory diseases (e.g., rheumatoid arthritis, tuberculosis, myelitis, malignancy, or liver/renal disease) in the elderly [30]. Poor appetite and decreased digestive function and/or digestive system diseases, reported as frequent co-morbidities in our study, also reflect poor nutrient digestion and absorption in the elderly. The “tea and toast lady syndrome”, a form of malnutrition in elderly subjects who are unable to prepare meals but nonetheless seek to feed themselves, can cause IDA [31]. The number of elderly subjects living alone increased 1.8-fold from 2005 to 2015. Elderly subjects who have seldom cooked by themselves, and who lack the ability to cook a nutritionally balanced menu, develop nutritional deficiencies including IDA. Since aging of Korea and amounts of elderly people living alone are increasing rapidly, supplying balanced meals to the elderly is very important to prevent and manage disease.

Notably, IDA was most common in females aged 40–49 years, followed by those aged 30–39 years. The higher prevalence of IDA in these age groups may be caused by pregnancy and postpartum issues. IDA accounts for about 75% of all cases of anemia during pregnancy; the iron supply is inadequate [32]. The maternal iron demand is 24 mg/day during the second part of pregnancy, but most Korean women commence pregnancy with minimal iron stores during the second trimester, with the average iron intake of 19.1 mg/day [33]. Untreated IDA during pregnancy is aggravated by blood loss during delivery, IDA can continue into the postpartum period, and indeed into the fifth decade of life [34]. Furthermore, IDA in females aged 30–39 and 40–49 is perhaps associated with abnormal uterine bleeding (AUB) accompanied by uterine myomas or endometrial polyps [35]. AUB is the most common cause of IDA in pre-menopausal women, and the risk increases in those aged ≥35 years [35,36]. The reason for the high rate of IDA prevalence in females aged 30–49 is expected to include pregnancy and breastfeeding, but more accurate classification of the causes is needed. Since the iron consumption or demand of this age group is different, it is a very important point of view for establishing policies for the prevention of IDA.

We present reliable data on the health expenditures imposed by IDA, i.e., both the direct medical and direct non-medical costs. These costs increased 2.4–2.9 fold over 12 years, probably attributable to an increase in IDA prevalence. Notably, outpatient costs increased continuously, being 6.2-fold those of hospitalization in 2013. Recent advances in health examination strategies allow detection of mild IDA treatable in outpatient settings.

IDA was accompanied by conditions of the respiratory and gastrointestinal tracts in both sexes. Iron status plays a key role in upper/lower respiratory tract infections and diseases such as the common cold, pneumonia, and asthma [37,38,39]. Although the reason is unclear, it is possible that IDA impairs the defense mechanisms of the airway mucosa [40]. Furthermore, gastrointestinal tract conditions may be accompanied by IDA because of increased iron loss (e.g., via melena, hematemesis, or rectal or invisible bleeding) and decreased iron absorption (e.g., in those with celiac disease or atrophic gastritis, and those of postsurgical status) [41,42]. Both physicians and IDA patients should be aware of the risks and act accordingly.

Our study had certain limitations. First, for the period 2002–2005, the NHIS database contains information on only NHI beneficiaries, and not MA beneficiaries. Therefore, IDA prevalence may have been underestimated [12]. There is another reason why the IDA prevalence rate is estimated to be low. The actual prevalence rate would have been underestimated as those who did not receive medical diagnosis or treatment were excluded from the claim. In other words, they may have been omitted from the diagnosis because they have already been treated or medicated and have not actually visited the hospital due to IDA. Second, we did not consider disease severity (as reflected by the hemoglobin level), although the costs associated with mild, moderate, and severe IDA differ. Third, we included only traffic cost of patient and caregiver for the estimation of direct non-medical cost, excluding caregiver’s economic costs for patient care. Therefore, economic cost might be underestimated. Finally, the analysis date was compliant with the data provision criteria of the Korea National Health Insurance Service, but it is currently somewhat old data, and biological observations have not been reflected. We need to maintain continuous trend analysis in the future. On the other hand, our research has differences and strengths compared to previous studies. Since the source of our data is Korean medical claim data, objectivity is secured, and the prevalence rate of IDA patients is calculated most accurately. This is the first nationwide study on the disease burden in Korea. In the case of Korea, an iron supplement support project is being implemented from a community health perspective, and the NutriPlus program, an iron-rich food delivery service, is being implemented for pregnant women, breastfeeding women, or infants. However, because these services are supported by economic income or by applicants only, not by iron deficiency, only some of them are eligible. To prevent and treat IDA, the government needs to make efforts to select more accurate targets and expand standards of support.

In conclusion, IDA remains a debilitating nutritional problem in terms of public health nutrition in Korea, the annual prevalence of which continues to rise in association with adverse health expenditures and co-morbidities. Infants and young children, the elderly, and females aged 30–49 years are at highest risk. A national, prospective and well-organized effort to improve iron status and nutrition monitoring systems to manage IDA are required.

## Figures and Tables

**Table 1:** Distribution in the sample cohort population and IDA-diagnosed patients, 2002–2013.

Variable	2002	2003	2004	2005	2006	2007	2008	2009	2010	2011	2012	2013
No. of Total population *												
All	1,025,340	1,017,468	1,016,580	1,016,820	1,002,005	1,020,743	1,000,785	998,527	1,006,481	1,011,123	1,014,730	1,014,730
Male	513,258	509,212	508,223	508,317	500,808	510,009	501,019	499,689	503,428	505,614	507,289	507,289
Female	512,082	508,256	508,357	508,503	501,197	510,734	499,766	498,838	503,053	505,509	507,441	507,441
No. of IDA population **												
All	3188	3806	4467	5049	5289	5730	6390	6575	6757	7286	7468	7325
Male	579	671	780	917	981	1047	1325	1298	1331	1493	1497	1523
Female	2609	3135	3687	4132	4308	4683	5065	5277	5426	5793	5971	5802

* Residence-registration mid-year population from Korean Statistical Information Service (KOSIS). ** Definition of IDA-diagnosed patients: subjects diagnosed as D50, D50.0, D50.8, or D50.9 as primary disease within that year.

**Table 2:** Prevalence of iron deficiency anemia in Korea, 2002–2013.

Variable	Age-Standardized Prevalence Rates per 100,000 * (%)														
	2002	2003	2004	2005	2006	2007	2008	2009	2010	2011	2012	2013	APC	(95% CI)	*p*-Value
No. of IDA patients (%)															
All	313.6	374.0	440.9	495.6	525.1	559.2	635.2	656.1	674.3	724.4	736.1	718.9	7.6	(5.9, 9.4)	<0.001
Male	117.7	134.7	157.0	180.7	197.4	204.6	261.6	258.2	265.5	296.6	291.7	293.9	8.9	(7.1, 10.8)	<0.001
Female	507.6	611.9	722.5	808.5	851.6	911.8	1008.5	1053.7	1083.7	1153.6	1182.9	1145.8	7.4	(5.6, 9.2)	<0.001
<10	188.9	279.0	306.5	415.2	494.4	610.0	885.5	1007.5	1057.9	1109.3	1173.1	1142.6	18.7	(14.6, 22.8)	<0.001
10–19	266.3	261.2	296.3	322.9	347.1	321.5	379.9	392.9	409.7	469.0	481.9	460.2	5.9	(4.9, 6.9)	<0.001
20–29	292.7	328.9	381.7	402.7	463.9	429.9	455.6	451.2	478.2	465.4	468.5	474.5	3.8	(2.1, 5.5)	<0.001
30–39	415.0	469.8	564.2	651.9	643.8	694.8	715.1	746.8	714.2	739.6	699.4	686.1	4.3	(2, 6.6)	0.002
40–49	405.2	555.6	652.8	708.7	768.8	827.9	949.1	934.3	991.2	1075.5	1067.5	1094.1	8.3	(6.1, 10.5)	<0.001
50–59	273.9	300.1	379.4	430.4	454.1	476.3	502.4	506.5	523.8	555.7	594.0	526.7	6.3	(4.2, 8.4)	<0.001
60–69	225.1	305.9	305.4	363.0	341.3	402.9	446.4	506.4	464.7	556.5	540.1	511.6	7.6	(5.6, 9.6)	<0.001
70–79	336.5	369.6	480.9	528.6	536.9	572.1	690.9	638.7	714.7	819.4	885.3	811.7	8.6	(6.8, 10.3)	<0.001
≥80	302.7	328.7	469.7	465.2	443.9	595.6	668.1	829.9	837.1	946.0	1149.7	1191.0	13.5	(11.7, 15.3)	<0.001
No. of IDA patients among males (%)															
<10	201.6	267.1	327.1	468.0	483.7	655.4	996.9	1048.3	1148.2	1193.1	1264.1	1209.5	19.1	(14.8, 23.6)	<0.001
10–19	149.1	130.7	124.0	172.4	182.6	156.8	187.9	140.1	174.7	191.6	159.3	168.4	2.0	(−0.4, 4.5)	0.098
20–29	34.7	58.2	65.2	71.4	89.2	55.6	95.3	89.6	91.6	118.7	78.6	95.9	7.2	(2.8, 11.8)	0.004
30–39	58.4	64.4	73.5	81.0	82.4	87.7	86.9	90.1	78.2	95.2	85.3	93.1	3.4	(1.7, 5.1)	0.001
40–49	74.7	83.6	109.5	93.9	130.2	101.7	123.8	112.6	104.9	151.0	127.6	147.0	4.9	(2.4, 7.6)	0.001
50–59	109.8	131.6	164.6	159.7	167.4	164.0	161.5	182.0	160.9	178.8	203.4	186.6	3.8	(1.9, 5.7)	0.001
60–69	197.9	226.3	242.9	272.6	247.0	313.8	367.9	389.5	363.5	428.3	415.2	413.8	7.4	(5.8, 9.1)	<0.001
70–79	311.6	369.8	422.2	475.5	587.4	550.4	660.9	557.4	593.3	693.0	647.3	679.3	6.6	(4.2, 9)	<0.001
≥80	341.4	324.4	512.8	532.1	573.2	633.8	841.4	922.9	884.7	682.7	1027.2	1010.2	10.5	(7.1, 14)	0.001
No. of IDA patients among females (%)															
<10	174.8	292.1	283.8	357.4	506.1	560.5	764.3	963.4	960.6	1019.3	1075.9	1071.3	18.2	(14.2, 22.3)	<0.001
10–19	395.6	406.5	489.3	491.8	532.6	506.7	595.0	676.9	672.9	778.7	841.1	783.4	7.2	(6, 8.3)	<0.001
20–29	556.3	605.1	703.6	742.5	848.8	815.8	831.1	829.8	886.9	836.3	889.1	887.5	3.8	(2.2, 5.4)	<0.001
30–39	787.7	890.6	1072.1	1241.4	1220.6	1320.9	1370.9	1430.9	1375.4	1408.0	1335.8	1303.2	4.3	(2, 6.7)	0.002
40–49	747.5	1048.7	1218.5	1351.4	1439.3	1582.3	1804.0	1784.5	1911.5	2038.7	2045.9	2072.5	8.5	(6.2, 10.9)	<0.001
50–59	435.9	467.8	595.4	700.5	740.9	789.8	847.1	834.9	889.1	934.5	987.7	872.5	6.9	(4.6, 9.3)	<0.001
60–69	247.8	373.8	359.0	441.3	424.2	482.0	516.9	612.5	557.9	675.4	656.9	603.4	7.9	(5.5, 10.3)	<0.001
70–79	350.9	369.5	516.5	561.7	505.1	586.2	711.2	695.0	800.2	910.0	1060.3	910.4	9.8	(7.9, 11.9)	<0.001
≥80	287.1	330.5	451.5	437.2	389.3	579.6	595.5	790.7	816.9	1057.2	1201.5	1268.0	14.7	(12.4, 17)	<0.001

APC, annual percent change; CI, confidence interval; IDA, iron deficiency anemia. * Adjusted to the sample cohort population in 2011.

**Table 3:** Female-to-male prevalence ratios of iron deficiency anemia in the sample cohort population, 2002–2013.

Variable	PRR														
	2002	2003	2004	2005	2006	2007	2008	2009	2010	2011	2012	2013	Slope	(95% CI)	*p*-Value
	No. of IDA patients (%)														
All	4.52	4.68	4.73	4.50	4.39	4.47	3.83	4.07	4.08	3.88	3.99	3.81	−0.08	(−0.11, −0.05)	<0.001
<10	0.87	1.09	0.87	0.76	1.05	0.86	0.77	0.92	0.84	0.85	0.85	0.89	−0.01	(−0.03, 0.01)	0.378
10–19	2.65	3.11	3.95	2.85	2.92	3.23	3.17	4.83	3.85	4.07	5.28	4.65	0.19	(0.08, 0.29)	0.003
20–29	16.01	10.39	10.80	10.40	9.52	14.68	8.73	9.26	9.68	7.05	11.31	9.25	−0.36	(−0.78, 0.06)	0.083
30–39	13.49	13.82	14.59	15.33	14.81	15.06	15.78	15.89	17.58	14.79	15.65	14.01	0.14	(−0.06, 0.33)	0.151
40–49	10.00	12.55	11.13	14.39	11.05	15.56	14.58	15.85	18.22	13.51	16.03	14.09	0.44	(0.09, 0.79)	0.02
50–59	3.97	3.55	3.62	4.39	4.43	4.82	5.25	4.59	5.52	5.23	4.86	4.68	0.13	(0.05, 0.21)	0.005
60–69	1.25	1.65	1.48	1.62	1.72	1.54	1.40	1.57	1.53	1.58	1.58	1.46	0.00	(−0.02, 0.03)	0.667
70–79	1.13	1.00	1.22	1.18	0.86	1.07	1.08	1.25	1.35	1.31	1.64	1.34	0.04	(0.01, 0.07)	0.016
≥80	0.84	1.02	0.88	0.82	0.68	0.91	0.71	0.86	0.92	1.55	1.17	1.26	0.04	(0.00, 0.08)	0.048

PRR, prevalence rate ratio; CI, confidence interval; IDA, iron deficiency anemia.

**Table 4:** Health Expenditures of iron deficiency anemia in Korea, 2002–2013.

Year	Category	Direct Medical Cost (①)			Direct Non-Medical Cost (②)		Total Cost	
		Paid by Insurer and Patients	Prescribed Pharmaceuticals (Hospital)	Prescribed Pharmaceuticals (Pharmacy)	Total Direct Medical Cost	Traffic Expense	Total Cost (① + ②)	Per Capita
	Outpatient	78,081	850	31,957	110,888	4760	115,648	42
2002	Hospitalization	71,137	4194	12,763	88,094	6461	94,555	100
	Total	149,218	5044	44,720	198,982	11,221	210,203	66
	Outpatient	89,058	2050	38,954	130,062	5978	136,040	43
2003	Hospitalization	94,958	5003	20,650	120,611	8781	129,392	101
	Total	184,017	7054	59,604	250,675	14,760	265,435	70
	Outpatient	111,484	2730	49,281	163,495	7640	171,135	46
2004	Hospitalization	124,980	9985	24,609	159,574	11,257	170,831	116
	Total	236,464	12,715	73,891	323,070	18,898	341,968	77
	Outpatient	137,784	2571	58,248	198,603	9316	207,919	48
2005	Hospitalization	148,801	10,436	24,223	183,460	12,198	195,658	126
	Total	286,585	13,008	82,472	382,065	21,514	403,579	80
	Outpatient	153,628	3997	67,726	225,351	10,517	235,868	52
2006	Hospitalization	152,398	9168	27,536	189,102	12,347	201,449	131
	Total	306,027	13,165	95,262	414,454	22,865	437,319	83
	Outpatient	188,227	9566	83,559	281,352	13,691	295,043	57
2007	Hospitalization	138,447	9040	19,244	166,731	9481	176,212	156
	Total	326,674	18,607	102,803	448,084	23,172	471,256	82
	Outpatient	207,482	7186	99,692	314,360	17,109	331,469	56
2008	Hospitalization	126,886	11,622	13,359	151,867	7576	159,443	199
	Total	334,368	18,808	113,052	466,228	24,686	490,914	77
	Outpatient	226,668	7983	101,786	336,437	17,975	354,412	58
2009	Hospitalization	128,694	7186	14,725	150,605	8127	158,732	186
	Total	355,363	15,170	116,512	487,045	26,103	513,148	78
	Outpatient	229,509	2788	108,159	340,456	18,623	359,079	57
2010	Hospitalization	131,289	8186	16,737	156,212	8386	164,598	201
	Total	360,799	10,974	124,896	496,669	27,009	523,678	78
	Outpatient	299,233	12,686	122,536	434,455	22,540	456,995	67
2011	Hospitalization	152,304	11,759	13,164	177,227	7835	185,062	240
	Total	451,538	24,445	135,700	611,683	30,375	642,058	88
	Outpatient	338,735	11,575	129,016	479,326	26,847	506,173	68
2012	Hospitalization	96,617	7167	34	103,818	1042	104,860	1008
	Total	435,353	18,743	129,050	583,146	27,890	611,036	82
	Outpatient	356,314	11,739	118,925	486,978	26,093	513,071	71
2013	Hospitalization	78,594	3576	7	82,177	1005	83,182	990
	Total	434,909	15,316	118,932	569,157	27,099	596,256	81

Unit: USD.
